# The Role of Fishing Piers in Brown Pelican (*Pelecanus occidentalis*) Entanglement

**DOI:** 10.3390/ani12182352

**Published:** 2022-09-08

**Authors:** Fairl L. Thomas, Elizabeth A. Forys

**Affiliations:** 1 Environmental Studies Discipline, Eckerd College, St. Petersburg, FL 33711, USA; 2 Environmental Studies and Biology Disciplines, Eckerd College, St. Petersburg, FL 33711, USA

**Keywords:** Brown Pelican, COVID-19 closure, entanglement, fishing pier, *Pelecanus occidentalis*

## Abstract

**Simple Summary:**

Brown Pelicans (*Pelecanus occidentalis*) are frequently entangled in fishing gear, particularly in areas with piers. Our study quantified the rate of pelican entanglement on four fishing piers in Tampa Bay, Florida, and measured variables we suspected might influence the number of entanglements. Over 7% of the Brown Pelicans seen near piers were entangled in fishing gear. Pelicans were more likely to be entangled at the two piers that had extensive perches near the piers. Reducing pelican perching near piers or reducing fishing near areas where pelicans perch would likely lead to a reduction in entanglement.

**Abstract:**

Throughout their range, Brown Pelicans *(Pelecanus occidentalis*) are one of the most common species to become entangled in fishing gear. We surveyed four piers every other week for one year (6/2019–5/2020) in the Tampa Bay region, FL, USA, to determine frequency of pelican entanglement associated with fishing piers, and explored factors that might influence the rate of entanglement. We conducted a generalized linear model (GLM) to determine the influence that pier, pier closure due to COVID-19, time of day and season, number of anglers, and presence of human behaviors that might attract pelicans to the pier had on the number of entangled pelicans. We conducted 144 surveys and counted 3766 pelicans of which 254 (7%) were entangled. The variables significantly associated (*p* < 0.05) with entanglement were the pier, time of day, and pier closure status, while the number and behavior of anglers were not significant. The two piers that most significantly influenced the number of entanglements both had extensive perches within 10 m of the fishing pier. The management action most likely to reduce the number of entangled pelicans appears to be deterring pelicans from perching near piers or decreasing fishing near perching structures.

## 1. Introduction

Entanglement in fishing gear is a major cause of injuries and death of seabirds [1,2,3,4,5]. Entanglement occurs when birds become hooked by or tangled in fishing gear most commonly while foraging [2,5]. While there are many types of debris that can result in entanglement, studies have found fishing related materials to be the most common cause of entrapment in marine animal species [6]. The Brown Pelican *(Pelecanus occidentalis*) is a large seabird which ranges along both the east and west coasts of North America and along the northern coasts of South America [7]. Researchers have determined that pelicans and other nearshore seabirds are at higher risk of entanglement in recreational fishing gear than their offshore counterparts because they are more likely to encounter anglers [2,8,9]. Additionally, the Brown Pelican’s large wingspan, flying and foraging method play a role in susceptibility to entanglement in active fishing gear [10,11]. Brown Pelican target prey from above, then plunge head first into the water to capture it [12,13]. Some studies indicate that fishing gear entanglement has been an increasing threat to the Brown Pelican since as early as the 1940s, noting it as an important cause of death in the species [1]. Brown Pelicans could encounter fishing gear from a number of sources: active fishing line cast by anglers off piers, bridges, shorelines or boats or improperly discarded fishing gear near perching or nesting areas, and in the water [5,14,15].

While the Brown Pelican has rebounded from past population declines due to reproductive failure from pesticide pollution and has been removed from the federal endangered species list [7,16], increasing mortality due to fishing line entanglement poses a risk to overall population stability [1,17]. In addition, the increase in rates of entanglement of pelicans poses a wildlife management and ethical concern [14,17].

There are relatively few studies on the role of fishing piers on Brown Pelican entanglement; however, it is widely known that Brown Pelicans are frequently injured on or near piers [17]. The number of injured pelicans could be due to the number of people fishing. Pelicans may be attracted to fishing piers due to anglers discarding food subsidies such as filleted fish and bait [14,18]; in addition, piers may provide attractive roosting locations [19]. Brown Pelicans can encounter fishing line while diving off piers for fish or simply by flying underneath piers where anglers are fishing [2,8].

Time of day and seasonal temperature changes could also influence the rates of entanglement at fishing piers. Pelicans are diurnal species that forage more in mornings, perhaps making it more likely for entanglement during this time period [20,21]. The highest reported mortality for juvenile and adult pelicans was in the cooler months of November [1]. This is likely because the colder weather results in a greater expenditure of energy to maintain body temperature, and high winds make it more difficult to locate and obtain prey, leading to higher mortality [22]. Hungry pelicans might be more apt to visit fishing piers where there could be discarded fish.

The objective of this paper is to quantify Brown Pelican entanglement at four major fishing piers in the Tampa Bay region, Florida (USA), and to determine if entanglement varies among different seasons, times of day, and when there are more people fishing. In addition, because the 2020 Covid-19-related closure of fishing piers occurred during our study, we are able to compare the number of entangled Brown Pelicans before, during, and after the closure.

## 2. Materials and Methods

**Figure 1:**
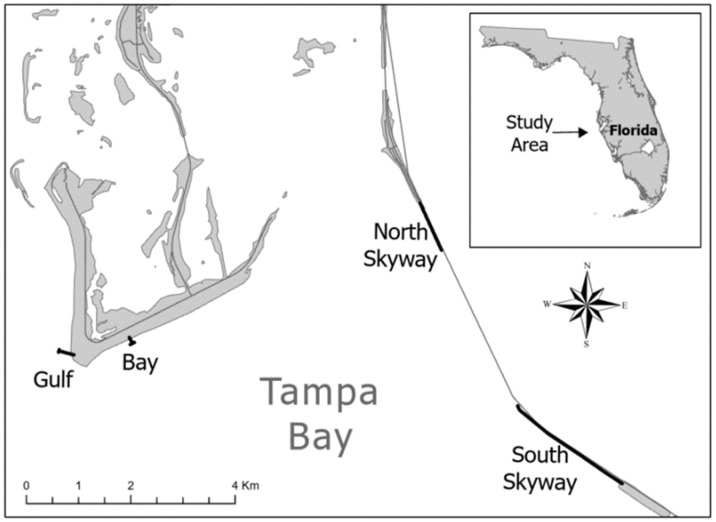
The four fishing piers located in the Tampa Bay region, Florida, USA. The Gulf and Bay piers are at Ft. De Soto County Park and the North and South Skyway piers are adjacent to the Sunshine Skyway Bridge.

Tampa Bay is located on the west coast of Florida adjacent to the Gulf of Mexico. This region serves as habitat for both migratory and year-round resident Brown Pelicans [1]. Four fishing piers in the Tampa Bay region, Florida, were chosen for observational surveying based on preliminary data that indicated they attracted a large number of anglers and Brown Pelicans. These included Bay Pier and Gulf Pier in Fort De Soto County Park, and North Skyway and South Skyway at Sunshine Skyway Fishing Pier State Park (Figure 1). All four piers were open to the public and did not require visitors to hold their own Florida state fishing license during the time of this study.

The Sunshine Skyway Fishing Piers State Park contains two fishing piers (North Skyway and South Skyway). In 1980, the Skyway Bridge, between the North and South Skyways, collapsed due to an accident. A new bridge was erected, and in 1987 some of the remnants of the old bridge were reopened as fishing piers, while other portions were destroyed [23]. The current North and South Skyway fishing piers are part of the original southbound lane. The South Pier (2816 m) is nearly twice the length of the North Pier (1328 m). Some of the original northbound lane exists, but is inaccessible to humans and serves as a predator-free roost for pelicans. The roosting area is much longer at the South Pier (1636 m) and much closer to the pier (7.6 m) compared to the North Pier’s roosting area, which is 341 m and 16.4 m apart. Both piers are 4.5 m above mean high tide.

The Florida Department of Environmental Protection began leasing the land and structures from the Florida Department of Transportation, opening the sites as a Florida state park in 1994. The Florida Park Service now manages these fishing piers, along with their contracted concessionaire, Pier Associates Incorporated. During the duration of our study, there were no Florida state park rangers stationed at the piers.

Fort De Soto County Park is a coastal island that contains the Gulf and Bay piers. The Bay Pier is located on the southwestern side of the island, jutting out into the waters of Tampa Bay. The Gulf Pier is located on the western side of the island, at the southernmost point, jutting out into the waters of the Gulf of Mexico. The Gulf Pier is significantly longer than the Bay Pier, extending to 308 m in length with a 71 m line of exposed rocks that pelicans use for perching, which is 8.6 m from the pier, whereas the Bay Pier is the shortest of the four piers (174 m) and only has a few pilings (3 m) usable as perches. These piers are 3.5 m above high tide.

We surveyed the four piers at least every other week for one year (6/2019–5/2020). Some of the piers were surveyed more frequently as part of another study, and all of the data were used in our analysis. At the start of each survey, we recorded the number of people actively fishing (e.g., holding or monitoring fishing gear) and the total number of Brown Pelicans on the pier, on an adjacent structure, or in the water within 3 m of the pier. Next, we walked down the pier and using 10x42 binoculars we closely observed the pelicans and recorded any that had signs of fishing line gear on their bodies and any dead pelicans. When possible, birds entangled with gear were captured and either untangled on site or transported to a licensed rehabber. On days when we were not surveying, other volunteers affiliated with local nonprofit wildlife rescue groups conducted rescue of entangled birds using similar methods, but these data were not included in our study. We also counted the number of times we saw activities that might attract pelicans and other birds to a pier (i.e., discarding filleted fish carcasses, used bait, or dead fish into the water).

The onset of the Covid-19 pandemic resulted in the closure of all public parks in April 2020, including our four fishing piers. We received special permissions from the Florida Department of Transportation, Florida Department of Environmental Protection, and Pinellas County Environmental Services in order to continue conducting surveys on a weekly basis at each of the study sites, despite these closures.

To understand which variables had a significant influence on the number of injured and dead Brown Pelicans (combined), we examined the data by constructing a generalized linear model (GLM) in SPSS 25.0 [24]. Because the number of injured and dead pelicans was count data and overdispersed (the variance was greater than the mean), our model used a negative binomial distribution with a log link function. Our independent variables included the pier, the total number of anglers, the season (warm season: May–October; cold season: November–April), time of day (morning: 7–10:49 am; mid-day: 11–1:59 pm; late afternoon 3–7 pm), Covid-19 pier closure (8 April–11 May 2020), and the number of live Brown Pelicans on the pier and nearby structures.

## 3. Results

From June 2019 through May 2020, a total of 144 surveys were conducted (Table 1). We observed 6054 anglers and 3766 Brown Pelicans at the four piers. Of the pelicans observed, 254 (7%) were identified as having signs of active fishing line entanglement or related injuries, and 17 were found dead.

**Figure 2:**
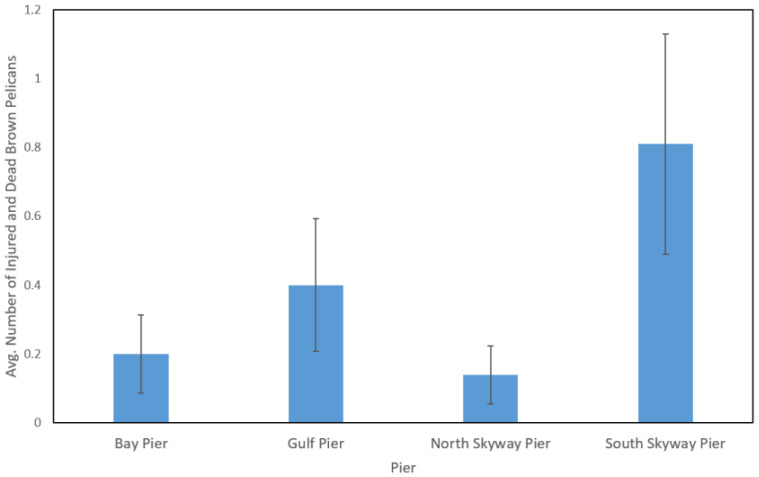
Estimated marginal mean (SE bars) number of injured and dead Brown Pelicans at the four piers for each survey after controlling other variables in the model. The South Skyway and Gulf piers had significantly more injured and dead pelicans than the Bay and North Skyway piers (*p* < 0.05, LSD post hoc test). Sample size = 144 surveys.

**Figure 3:**
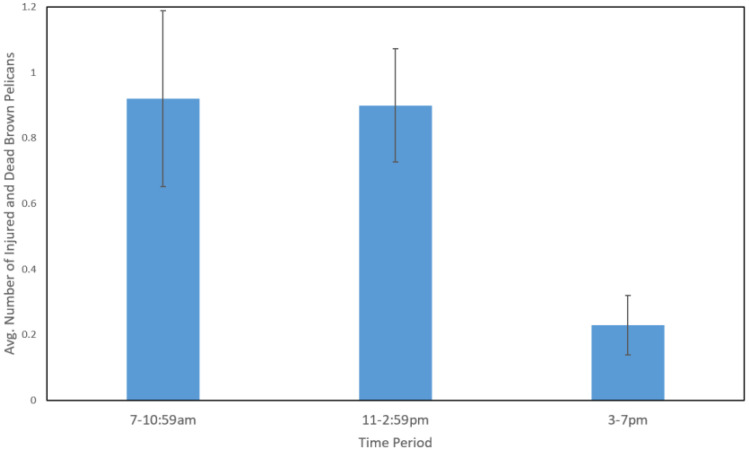
Estimated marginal mean (SE bars) number of injured and dead Brown Pelicans during three times of day after controlling for the other variables in the model. The 7–10:59 am and 11–2:59 pm time periods had significantly more injured and dead pelicans than the 3–7 pm time period (*p* < 0.05, LSD post hoc test). Sample size = 144 surveys.

The generalized linear model explained a significant amount of variation in the data (likelihood ratio χ^2^ = 137.8, d.f. = 12, *p* < 0.0001). Pier (χ^2^ = 12.493. d.f. = 3, *p* = 0.006), time of day (χ^2^ = 11.922, d.f. = 2, *p* = 0.003), and pier closure (χ^2^ = 7.313, d.f. = 1, *p* = 0.007) were the significant independent variables (Table 2). The South Skyway and Gulf piers had significantly more injured and dead pelicans (after factoring in the influence of the other variables in the model) than the Bay and North Skyway piers (Figure 2). Significantly more pelicans were injured in the morning and mid-day hours compared to the afternoon (Figure 3). While most of our surveys were conducted when the piers were open to fishing (*n* = 128), we were able to do 16 surveys when the piers were closed due to Covid-19. During these 16 surveys, we found 3 injured or dead pelicans (mean ± SD = 0.19 ± 0.4) compared to 251 seen during the times when the pier was open to fishing (mean ± SD = 2.09 ± 3.68). Number of anglers on the pier, number of pelicans, and all three of the behaviors that might attract pelicans to piers (discarding of filleted carcasses, dead fish, and bait) were not significant. Season was not statistically significant, but approached significance (*p* = 0.053). 

## 4. Discussion

This study found that recreational fishing from public piers poses a large threat to Brown Pelicans in Tampa Bay, as >7% of pelicans observed had signs of entanglement or related injuries. Without constant monitoring and intervention by volunteers, many of these pelicans could die. The number of pelicans and anglers on a pier did not significantly affect the number of injured and dead pelicans counted during a survey; instead, the individual pier was the most significant variable. When other variables were held constant, pelicans surveyed near the South Skyway Pier and the Gulf Pier at Ft. De Soto were significantly more likely to be entangled with fishing gear compared to pelicans seen near the North Skyway Pier and Bay Pier at Ft. De Soto.

One potential explanation for these significant differences in the piers could be that anglers at the South Skyway and Gulf piers were engaging in more behaviors that attract pelicans to areas near recreational fishing activity. However, none of these behaviors (discarding of carcasses, dead fish, and bait) were significantly associated with entanglement and, at all the piers, the frequency of these behaviors was very low (Table 2). This could be due to previous and ongoing efforts to educate anglers about the threats that these activities pose to birds [25,26]. Similar efforts were successful in other parts of Florida [17].

Anecdotal observations (Thomas, pers. obs.) about how pelicans become entangled points toward the physical proximity of perches to the area with the most active fishing. The South Skyway Pier is 2816 m in length and running parallel to it is 1636 m of an old bridge expanse that is 7.6 m from the pier, which is the primary area where Brown Pelicans perch at this location. When these birds leave their perch to fish, they often run into active fishing lines and become entangled. A similar situation exists at the Gulf Pier at Ft. De Soto, where much of the fishing on the 378 m pier is concentrated near a 71 m large rock jetty that runs near (8.6 m) and parallel to the pier. The adjacent perching areas at the North Skyway Pier are shorter than at the South Skyway Pier, and are nearly double the distance from the pier (16.4 m). At the Bay Pier at Ft. De Soto, there are few areas for pelicans to perch adjacent to the pier. Other researchers have hypothesized that the plunge diving habit of Brown Pelicans puts them at particular risk for entanglement [2,9].

The theory that pelicans are more likely to be entangled when pelicans leave a perch close to a pier is further supported by the two other significant variables: time of day and the Covid-19 closure. Pelicans forage significantly more during the morning and if entanglement is associated with pelicans foraging, then it makes sense that this variable is significant [20,21,27]. Our surveys found more entangled pelicans both during the morning and mid-day surveys than late afternoon. We recorded the number of entangled birds seen during the survey, not when the actual entanglement occurred. It is likely that entangled birds from the mornings were being seen during our mid-day surveys.

In addition, surveys conducted during the Covid-19 park closures recorded high numbers of Brown Pelicans, but virtually no entanglements. During the time in which the piers were closed, anglers were still permitted to fish from boats, and there was a surge in recreational boating [28]. This indicates pier fishing may increase the risk of entanglement more than boat fishing. Research in Naples, FL, showed a similar result; most of the entangled birds came from an area around a major fishing pier [17]. This increased risk of entanglement at piers may be, in part, due to the elevated nature of pier structures, which allows birds to fly underneath anglers, which can be in direct path through active lines hanging from above. Owing to the thin and sometimes transparent materials that comprise fishing lines, the birds are extremely unlikely to recognize the presence of line in time to change their course of flight [29]. We believe this causes fishing from piers to pose a greater threat than recreational fishing from vessels because anglers are more dispersed amongst a greater area, and active lines are more level with the water’s surface when fishing from a boat.

It was somewhat surprising that the cold season (November–April) was not significantly associated with more entanglements, based on data that shows Brown Pelicans have an increased need for food [22]. However, the average cold season temperatures during our field work in Pinellas County were significantly higher than average, and March 2020 was the warmest on record (National Weather Service Data for Pinellas County, Pinellas County, FL, USA).

## 5. Conclusions

Our study demonstrates that Brown Pelican entanglement is a significant problem. The management actions most likely to reduce the number of entangled pelicans appear to be removing perches close to piers, deterring pelicans from perching close to piers, or decreasing fishing near the areas where pelicans perch. After the conclusion of our study, managers from Ft. De Soto closed to fishing the portion of the Gulf Pier near the rocks where the pelicans perched on a seasonal basis. This resulted in a dramatic decline in entangled birds at this site during the cold season. Further research should involve how and when pelicans are entangled and seek creative solutions for removing adjacent perches or deterring birds from using these perches. Ongoing angler interviewing and education about reducing activities that attract pelicans and what to do when you hook a pelican is also important and has worked with other species [30,31].

## Figures and Tables

**Table 1:** Variables measured during each Tampa Bay, Florida, pier survey (mean ± SD).

Variables	Bay (*n* = 36)	Gulf (*n* = 45)	North Skyway (*n* = 23)	South Skyway (*n* = 40)
Entangled pelicans (injured+dead)	0.44 ± 1.08	1.09 ± 2.34	0.30 ± 0.47	4.97 ± 4.92
Pelicans	5.17 ± 7.66	14.44 ± 12.95	27.61 ± 23.72	57.38 ± 52.73
Anglers	11.33 ± 17.90	26.38 ± 16.30	47.83 ± 31.05	83.98 ± 67.10
Discarded fish carcasses	0.44 ± 1.73	0.47 ± 1.55	0.22 ± 0.85	0.35 ± 0.95
Discarded dead fish	0.00 ± 0.00	0.73 ± 1.36	0.35 ± 0.78	1.45 ± 1.71
Discarded bait	0.44 ± 1.08	1.09 ± 2.34	0.30 ± 0.47	4.97 ± 4.92

**Table 2:** Significance of factors predicting injured and dead Brown Pelicans using a general linear model. DF = degrees of freedom; Sig. = statistical significance (*p*).

Source	Type III Wald Chi Square	DF	Sig.
(Intercept)	0.388	1	0.533
Pier	12.493	3	0.006
Anglers	0.024	1	0.876
Pelicans	2.974	1	0.085
Discarded carcasses	2.915	1	0.088
Discarded dead fish	1.913	1	0.167
Discarded bait	0.534	1	0.465
Season	3.785	1	0.052
Time of day	11.922	2	0.003
Covid-19 closure	7.313	1	0.007

## Data Availability

Data are available upon request from the primary author.
